# Lack of epistatic interaction of *SNCA* with *APOE* in synucleinopathies

**DOI:** 10.1093/braincomms/fcaf455

**Published:** 2025-11-17

**Authors:** Prabhjyot Saini, Eric Yu, Mehrdad A Estiar, Lynne Krohn, Kheireddin Mufti, Uladzislau Rudakou, Jennifer A Ruskey, Farnaz Asayesh, Sandra B Laurent, Dan Spiegelman, Isabelle Arnulf, Jacques Y Montplaisir, Jean-François Gagnon, Alex Desautels, Yves Dauvilliers, Gian Luigi Gigli, Mariarosaria Valente, Francesco Janes, Andrea Bernardini, Karel Šonka, David Kemlink, Wolfgang H Oertel, Karri Kaivola, Annette Janzen, Giuseppe Plazzi, Elena Antelmi, Francesco Biscarini, Michela Figorilli, Monica Puligheddu, Brit Mollenhauer, Claudia Trenkwalder, Friederike Sixel-Döring, Valérie Cochen De Cock, Christelle Charley Monaca, Anna Heidbreder, Luigi Ferini-Strambi, Femke Dijkstra, Mineke Viaene, Beatriz Abril, Bradley F Boeve, Ronald B Postuma, Guy A Rouleau, Victoria Anselmi, Abubaker Ibrahim, Ambra Stefani, Birgit Högl, Michele T M Hu, Sonja W Scholz, Ziv Gan-Or

**Affiliations:** Montreal Neurological Institute, McGill University, Montreal, Quebec, Canada H3A 1A1; Department of Human Genetics, McGill University, Montreal, Quebec, Canada H3A 1Y2; Montreal Neurological Institute, McGill University, Montreal, Quebec, Canada H3A 1A1; Department of Human Genetics, McGill University, Montreal, Quebec, Canada H3A 1Y2; Montreal Neurological Institute, McGill University, Montreal, Quebec, Canada H3A 1A1; Department of Human Genetics, McGill University, Montreal, Quebec, Canada H3A 1Y2; Broad Institute of Massachusetts Institute of Technology and Harvard University, Cambridge, MA 02142, USA; Montreal Neurological Institute, McGill University, Montreal, Quebec, Canada H3A 1A1; Department of Human Genetics, McGill University, Montreal, Quebec, Canada H3A 1Y2; Montreal Neurological Institute, McGill University, Montreal, Quebec, Canada H3A 1A1; Department of Human Genetics, McGill University, Montreal, Quebec, Canada H3A 1Y2; Montreal Neurological Institute, McGill University, Montreal, Quebec, Canada H3A 1A1; Department of Human Genetics, McGill University, Montreal, Quebec, Canada H3A 1Y2; Montreal Neurological Institute, McGill University, Montreal, Quebec, Canada H3A 1A1; Department of Neurology and Neurosurgery, McGill University, Montreal, Quebec, Canada H3A 2B4; Montreal Neurological Institute, McGill University, Montreal, Quebec, Canada H3A 1A1; Department of Neurology and Neurosurgery, McGill University, Montreal, Quebec, Canada H3A 2B4; CHU Sainte Justine, University of Montreal, Montreal, Quebec, Canada H3T 1C5; Montreal Neurological Institute, McGill University, Montreal, Quebec, Canada H3A 1A1; Department of Neurology and Neurosurgery, McGill University, Montreal, Quebec, Canada H3A 2B4; Sleep Disorders Unit, Pitié-Salpêtrière Hospital, Centre de Recherche de l’Institut du Cerveau et de la Moelle Épinière, and Sorbonne Universités, Paris 75013, France; Centre d’Études Avancées en Médecine du Sommeil, Hôpital du Sacré-Cœur de Montréal, Montreal, Quebec, Canada H4J 1C5; Department of Psychiatry, Université de Montréal, Montreal, Quebec, Canada H3T 1J4; Centre d’Études Avancées en Médecine du Sommeil, Hôpital du Sacré-Cœur de Montréal, Montreal, Quebec, Canada H4J 1C5; Department of Psychology, Université du Québec à Montréal, Montreal, Quebec, Canada H2X 3JB; Centre d’Études Avancées en Médecine du Sommeil, Hôpital du Sacré-Cœur de Montréal, Montreal, Quebec, Canada H4J 1C5; Department of Neurosciences, Université de Montréal, Montreal, Quebec, Canada H3C 3J7; National Reference Center for Narcolepsy, Sleep Unit, Department of Neurology, Gui-de-Chauliac Hospital, Centre Hospitalier Universitaire Montpellier, University of Montpellier, INSERM U1061, Montpellier 34090, France; Clinical Neurology Unit, Department of Head and Neck, University Hospital of Udine, Udine 33100, Italy; Department of Medicine (DMED), University of Udine, Udine 121 08, Italy; Clinical Neurology Unit, Department of Head and Neck, University Hospital of Udine, Udine 33100, Italy; Department of Medicine (DMED), University of Udine, Udine 121 08, Italy; Clinical Neurology Unit, Department of Head and Neck, University Hospital of Udine, Udine 33100, Italy; Clinical Neurology Unit, Department of Head and Neck, University Hospital of Udine, Udine 33100, Italy; Department of Neurology and Centre of Clinical Neuroscience, First Faculty of Medicine and General University Hospital, Charles University, Prague 121 08, Czech Republic; Department of Neurology and Centre of Clinical Neuroscience, First Faculty of Medicine and General University Hospital, Charles University, Prague 121 08, Czech Republic; Department of Neurology, Philipps University Marburg, Marburg 35032, Germany; Neurodegenerative Diseases Research Section, National Institute of Neurological Disorders and Stroke, Bethesda, MD 20892, USA; Department of Neurology, Philipps University Marburg, Marburg 35032, Germany; Department of Biomedical and Neuromotor Sciences (DIBINEM), Alma Mater Studiorum—University of Bologna, Bologna 40126, Italy; IRCCS—Institute of Neurological Sciences of Bologna, Bologna 40139, Italy; Department of Engineering and Medicine of Innovation (DIMI), University of Verona, Verona 37134, Italy; Department of Biomedical and Neuromotor Sciences (DIBINEM), Alma Mater Studiorum—University of Bologna, Bologna 40126, Italy; Department of Medical Sciences and Public Health, Sleep Disorder Research Center, University of Cagliari, Cagliari 09042, Italy; Department of Medical Sciences and Public Health, Sleep Disorder Research Center, University of Cagliari, Cagliari 09042, Italy; Paracelsus-Elena-Klinik, Kassel 34128, Germany; Department of Neurology, University Medical Center Göttingen, Göttingen 37075, Germany; Paracelsus-Elena-Klinik, Kassel 34128, Germany; Department of Neurology, University Medical Center Göttingen, Göttingen 37075, Germany; Department of Neurology, Philipps University Marburg, Marburg 35032, Germany; Paracelsus-Elena-Klinik, Kassel 34128, Germany; Sleep and Neurology Unit, Beau Soleil Clinic, Montpellier 34070, France; EuroMov Research Laboratory, University of Montpellier, Montpellier 34090, France; Department of Clinical Neurophysiology and Sleep Center, University of Lille Nord de France, Centre Hospitalier Universitaire Lille, Lille 59045, France; Department of Neurology and Clinical Research Institute for Neuroscience, Johannes Kepler University Linz, Linz 4020, Austria; Department of Neurological Sciences, Università Vita-Salute San Raffaele, Milan 20132, Italy; Laboratory for Sleep Disorders, Sint-Dimpna Regional Hospital, Geel 2440, Belgium; Department of Neurology, Sint-Dimpna Regional Hospital, Geel 2440, Belgium; Department of Neurology, University Hospital Antwerp, Edegem, Antwerp 2650, Belgium; Laboratory for Sleep Disorders, Sint-Dimpna Regional Hospital, Geel 2440, Belgium; Department of Neurology, Sint-Dimpna Regional Hospital, Geel 2440, Belgium; Sleep Disorder Unit, Carémeau Hospital, Centre Hospitalier Universitaire Nîmes, Nîmes 30029, France; Department of Neurology, Mayo Clinic, Rochester, MN 55905, USA; Montreal Neurological Institute, McGill University, Montreal, Quebec, Canada H3A 1A1; Department of Neurology and Neurosurgery, McGill University, Montreal, Quebec, Canada H3A 2B4; Montreal Neurological Institute, McGill University, Montreal, Quebec, Canada H3A 1A1; Department of Human Genetics, McGill University, Montreal, Quebec, Canada H3A 1Y2; National Reference Center for Narcolepsy, Sleep Unit, Department of Neurology, Gui-de-Chauliac Hospital, Centre Hospitalier Universitaire Montpellier, University of Montpellier, INSERM U1061, Montpellier 34090, France; Sleep Disorders Clinic, Department of Neurology, Medical University of Innsbruck, Innsbruck 6020, Austria; Sleep Disorders Clinic, Department of Neurology, Medical University of Innsbruck, Innsbruck 6020, Austria; Sleep Disorders Clinic, Department of Neurology, Medical University of Innsbruck, Innsbruck 6020, Austria; Oxford Parkinson’s Disease Centre, University of Oxford, Oxford OX1 3QX, UK; Nuffield Department of Clinical Neurosciences, University of Oxford, Oxford OX3 9DU, UK; Neurodegenerative Diseases Research Section, National Institute of Neurological Disorders and Stroke, Bethesda, MD 20892, USA; Department of Neurology, Johns Hopkins University School of Medicine, Baltimore, MD 21287, USA; Montreal Neurological Institute, McGill University, Montreal, Quebec, Canada H3A 1A1; Department of Human Genetics, McGill University, Montreal, Quebec, Canada H3A 1Y2; Department of Neurology and Neurosurgery, McGill University, Montreal, Quebec, Canada H3A 2B4

**Keywords:** Parkinson’s disease, synucleinopathies, dementia with Lewy bodies, REM sleep behaviour disorder, epistasis

## Abstract

Two recent studies suggested that the *APOE* ε4 haplotype was associated with increased α-synuclein pathology in cell and mouse models. Genetic variants in the *SNCA* region have strong association with Parkinson’s disease (PD), dementia with Lewy bodies (DLB) and idiopathic REM sleep behaviour disorder (iRBD), while *APOE* is a genetic risk determinant for only DLB. To determine if genetic-level interactions between *SNCA* and *APOE* exists that can explain the protein-level association, we investigated the genotypic interaction of *APOE* and *SNCA* in cohorts of PD, DLB and iRBD. We analysed genome-wide association study (GWAS) data from 5229 PD patients and 5480 controls, 2610 DLB patients and 1920 controls, and 1055 iRBD patients and 3667 controls. We used logistic regression interaction models across all three cohorts independently between the (i) top GWAS signals of *SNCA* single nucleotide polymorphisms (SNPs) and *APOE* haplotypes and (ii) SNP×SNP and three-way SNP interaction across the entire coding region plus 200 kb flanking each gene. No significant interactions were found to be associated with any of the synucleinopathies after correction for multiple testing. Our results do not support a role for genetic interactions between *APOE* and *SNCA* across PD, DLB and iRBD. Since the tested genetic variants affect the expression and function of these proteins, it is likely that any interactions between them do not affect the risk of PD, DLB and iRBD.

## Teaser

Saini *et al.* report no evidence of genetic interaction between *SNCA* and *APOE* in 8855 synucleinopathy patients and 11 067 controls. The authors found no evidence of genetic epistasis between these genes, suggesting that protein-level interactions between *APOE* and *SNCA* are unlikely to be driven by synucleinopathy risk variants.

## Graphical Abstract

**Graphical Abstract:**
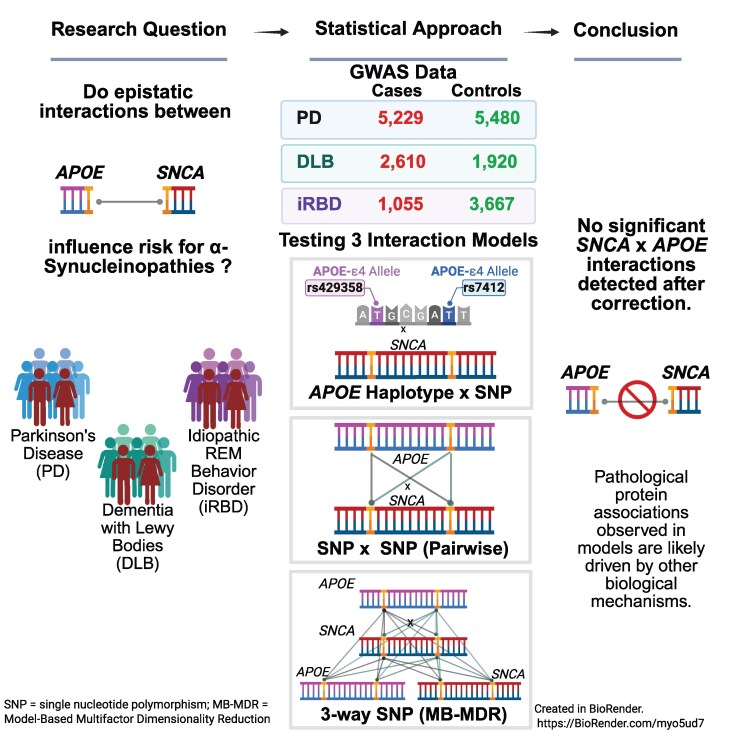

## Introduction

Alpha-synucleinopathy is an umbrella term to describe several neurodegenerative diseases that have a common defining pathological feature, characterized by neuronal or glial inclusions of aggregated alpha-synuclein, known as Lewy bodies, Lewy neurites or glial cytoplasmatic inclusions in the brain.^1^ Disorders that are collectively referred to as alpha-synucleinopathies include Parkinson’s disease (PD), dementia with Lewy bodies (DLB) and multiple system atrophy (MSA). Furthermore, there is an increased presence of alpha-synucleinopathy in the prodromal condition idiopathic/isolated REM sleep behaviour disorder (iRBD), which can convert to either PD, DLB or MSA in more than 80% of cases.^2^

Alpha-synuclein is encoded by the *SNCA* gene, and genetic variants in the *SNCA* locus are associated with PD, DLB and iRBD risk in genome-wide association studies (GWASs).^3-8^ Specifically, some variants of *SNCA* are strongly associated with PD (rs356182 and rs2870004), while others are associated with DLB (rs7681440 and rs7680557)^7,9^ and iRBD (rs2870004).^10^ The top *SNCA* association in PD is independent and different than the top associations in DLB^7^ and iRBD,^8^ raising the hypothesis that there could be differential effects of *SNCA* variants on the expression of alpha-synuclein in different brain regions.^8^

Overlapping neuropathologic features associated with Alzheimer’s disease (AD) are seen in the brains of many patients with PD^11^ and dementia including amyloid plaques composed of amyloid-beta (Aβ) plaques and neurofibrillary tangles containing the tau protein and may contribute to clinical features of disease.^12,13^

Coding variants in apolipoprotein E (*APOE*) produce 3 common alleles, ε2, ε3 and ε4. The *ε4* allele of *APOE* alters lipid metabolism regulation and cholesterol transport and known to be a major genetic risk determinant for sporadic, late-onset Alzheimer’s disease^14^ and Lewy body dementia.^7,9^ Dose effects by allele have demonstrated a 3.7-fold risk of developing AD while homozygosity increases the risk by up to 12-fold.^7^ From numerous GWASs, A*POE* does not alter the risk for PD, yet the ε4 allele has been described as a potential risk factor for cognitive decline and development of dementia in PD patients.^15,16^

Two studies in mice demonstrated that the APOE*ε4 genotype was associated with increased alpha-synuclein pathology, independent of the amyloid β deposition.^17,18^ These two studies emphasize a potential molecular mechanism of APOE*ε4 on α-synuclein protein aggregation. However, beyond A53T, they have not evaluated disease specific variants of *SNCA* that have functional molecular consequences (e.g. E46K).^19^ Analysis of these variants could be insightful in understanding molecular association between *SNCA* variants and APOE*ε4. Furthermore, while the *SNCA* locus is associated with all synucleinopathies, *APOE* is a genetic risk factor for DLB only. *SNCA* and *APOE* variants may affect the expression/function of the proteins encoded by them.^20^ Therefore, if a true interaction exists at the protein level as suggested by the studies mentioned above, then there plausibly should be evidence of some association at the genetic level.

Genetic interactions refer to a combination of two or more genetic variants whose phenotypic contribution is amplified by their co-occurrence.^21^ To determine if genetic-level interactions between *SNCA* and *APOE* exist that can explain the protein-level association as described, we investigated the genotypic interaction of *APOE* and *SNCA* in three disease cohorts of PD, DLB and iRBD patients and controls, with a total of 8855 patients and 11 067 controls.

## Materials and methods

### Patient population

For PD, we used the International Parkinson’s Disease Genomics Consortium (IPDGC) dataset that contained 10 709 subjects with 5229 cases and 5480 controls. For DLB, we used the most recent DLB GWAS dataset, which included 4530 subjects with 2610 cases and 1920 controls. The iRBD cohort was composed of 4742 individuals, including 1055 cases and 3667 controls with 1968 controls from the NeuroGenetics Research Consortium (NGRC) (dbGAP: phs000196.v2.p1) and 790 controls from National Institute of Neurological Disorders and Stroke (NINDS) (dbGAP: phs000089) added from external studies of Parkinson’s patients in addition to the controls collected for the iRBD cohort. PD was diagnosed using UK Brain Bank criteria or Movement Disorders Society (MDS) criteria.^4^ DLB patients were diagnosed with pathologically definite or clinically probable disease according to consensus criteria.^22^ iRBD was diagnosed according to the International Classification of Sleep Disorders (2nd or 3rd Edition).^23,24^ Informed consent and ethics approval was obtained from the appropriate institutional review boards at participating institutions as described in the original studies. The STREGA reporting guidelines were used for this study.^17^

### Genetic analysis

We generated genotype calls of two *APOE* SNPs, rs429358 and rs7412, to determine the *APOE* haplotype status of each sample. The combination of genotypes for rs429358 (C/T) and rs7412 (C/T) defines the three *APOE* haplotypes: epsilon 2 (ε2), epsilon 3 (ε3) and epsilon 4 (ε4). These three haplotypes can produce six genotypes, ε2/ε2, ε2/ε3, ε2/ε4, ε3/ε3, ε3/ε4 and ε4/ε4.

Cohorts were collected, quality controlled, genotyped, and filtered on individual and variant level as previously described for PD,^4^ iRBD^8^ and DLB.^7^  *APOE* variants were analysed for the 200 kb region flanking both sides on chromosome 19, 44 705 791–45 109 393, and for *SNCA* on chromosome 4, 89 500 345–90 038 324. The DLB genotypic data was converted from GrCh38 to GrCh37 using Liftover.^18^ Because external controls were added to the iRBD cohort; the cases and control genotypes were filtered for minor allele frequency (MAF) > 0.01 to reduce imputation errors and imputed using Michigan Imputation Server and the Haplotype Reference Consortium^19^ r1.1 2016 reference panel (GRCh37/hg19). Only imputed genotypes with an *R*^2^ > 0.30 were kept for analysis. Additionally, prior to analysis, further quality control for each cohort included removing duplicate samples, missing data including covariates; SNPs were filtered based on variant missingness (<0.05), genomic relatedness (>0.125), disparate missingness between cases and controls (*P* > 1E-04), missingness by haplotype (*P* > 1E-04), deviation from Hardy–Weinberg equilibrium (*P* > 1E-04), minor allele frequency (MAF) > 0.01 and LD pruned with r^2^ at >0.5 with a 50 kb window using plink 1.9.^20^

### Statistical analysis

Descriptive measures of mean, standard deviations, frequencies and percentages were used to summarize the data. SNP and haplotype interaction were analysed using logistic regression controlling for age, sex and ancestry using the first five principal components. Epistasis model was defined as


$$Y\;∼\;\beta_{0}+\beta_{1}A+\beta_{2}B+\beta_{3}AB+\epsilon$$

where $\beta_{0}$ represents the intercept, $\beta_{n}$ represents the coefficients for each SNP and A and B represent allele dosage of each SNP and AB represents the interaction. The test for interaction was based on the coefficient $\beta_{3}$ and *P*-value < 0.05 as significant when testing interaction between top SNPs and Bonferroni threshold applied for SNP×SNP interactions. Detecting gene–gene interactions in complex diseases can be accomplished using a variety of epistasis-focused tools or packages in existing software packages. Model-based multifactor dimensionality reduction (MB-MDR) was implemented as a final screen strategy to detect any significant SNP×SNP and SNP×SNP×SNP interactions using the open-source MB-MDR v 4.4.1 software, as MB-MDR merges multi-locus genotypes exhibiting some significant evidence of High or Low risk, based on association testing into a new lower-order dimension. A new association test is subsequently performed per marker pair/triplet, by adopting a permutation-based strategy that corrects for multiple testing (over all marker pairs/triplets) and adequately controls family-wise error rate at *α* = 5%.^21^

## Results

To explore possible interactions between *APOE* and *SNCA* in cases and controls, we first focused primarily on the top *SNCA* SNPs associated with PD, DLB and RDB (Table 1) and the *APOE* haplotypes (Table 2). We performed a SNP–haplotype logistic regression interaction controlling for age, sex and ancestry using the first five principal components. No significant interactions were found to be statistically significant (Table 3).

**Table 1:** Age and sex of each cohort in cases and controls

Demographic			Total	PD	DLB	iRBD
Sex	*Cases*	*Male (%)*	5845 (65.72)	3366 (64.37)	1642 (62.91)	837 (79.34)
		*Female (%)*	3010 (33.84)	1863 (35.63)	947 (36.28)	200 (18.96)
	*Controls*	*Male (%)*	7256 (65.56)	3055 (55.75)	972 (50.63)	1564 (42.65)
		*Female (%)*	3811 (34.44)	2425 (44.25)	948 (49.38)	2103 (57.34)
Age	*Cases*		65.7 (13.6)	61.2 (12.6)	74.7 (10.5)	-
	*Controls*		67.9 (16.2)	64.3 (14.8)	72.7 (16.8)	-

Values for age represent mean and standard deviation in parenthesis. Age information for iRBD was missing.

Abbreviations: PD, Parkinson’s disease; DLB, dementia with Lewy bodies; iRBD, idiopathic rapid eye movement (REM) sleep behaviour disorder.

**Table 2:** APOE haplotype frequencies of each cohort in cases and controls

Group	Haplotype	Controls	Cases	Total
DLB	e2e2	4 (0.21)	5 (0.19)	9
	e2e3	171 (8.91)	189 (4.17)	360
	e2e4	17 (0.89)	83 (3.18)	100
	e3e3	1273 (66.30)	1203 (46.09)	2476
	e3e4	419 (21.82)	916 (35.10)	1335
	e4e4	36 (1.88)	214 (8.20)	250
	**Total**	**1920** (**42.38)**	**2610** (**57.62)**	**4530**
iRBD	e2e2	22 (0.61)	5 (0.48)	27
	e2e3	444 (12.27)	109 (10.4)	553
	e2e4	82 (2.27)	15 (1.43)	97
	e3e3	2241 (61.92)	671 (64.03)	2912
	e3e4	752 (20.78)	236 (22.52)	988
	e4e4	78 (2.16)	12 (1.15)	90
	**Total**	**3619** (**77.54)**	**1048** (**22.46)**	**4667**
PD	e2e2	41 (0.75)	25 (0.48)	66
	e2e3	725 (13.23)	656 (12.55)	1381
	e2e4	106 (1.93)	75 (1.43)	181
	e3e3	3599 (65.68)	3498 (66.90)	7097
	e3e4	954 (17.41)	932 (17.82)	1886
	e4e4	55 (1.00)	43 (0.82)	98
	**Total**	**5480** (**51.17)**	**5229** (**48.83)**	**10709**

Values represent frequency and percentage. Bold values represent totals.

Abbreviations: PD, Parkinson’s disease; DLB, dementia with Lewy bodies; iRBD, idiopathic rapid eye movement (REM) sleep behaviour disorder.

**Table 3:** Top hits of SNCA and APOE haplotype interaction regression results

Disease	Gene	Chr	Location	Ref Allele	Alt Allele	rs	Interaction	Estimate	Standard Error	*z*-score	Pr(>|z|)
iRBD	*SNCA*	*4*	90471245	T	A	rs2870004	rs2870004×*APOE* Haplotype	−0.06359	0.06976	−0.912	0.362
		*4*	90626111	G	A	rs356182	rs356182×*APOE* Haplotype	0.03883	0.0629	0.617	0.537021
		*4*	90756550	C	G	rs7681440	rs7681440–*APOE* Haplotype	0.04838	0.05954	0.812	0.416257
PD	*SNCA*	*4*	90471245	T	A	rs2870004	rs2870004×*APOE* Haplotype	0.039526	0.038801	1.019	0.30835
		*4*	90626111	G	A	rs356182	rs356182×*APOE* Haplotype	0.027522	0.031979	0.861	0.389447
		*4*	90756550	C	G	rs7681440	rs7681440–*APOE* Haplotype	−0.016695	0.030556	−0.546	0.584807
DLB	*SNCA*	*4*	89550094	T	A	rs2870004	rs2870004×*APOE* Haplotype	−0.139925	0.118107	−1.185	0.2361
		*4*	89704960	G	A	rs356182	rs356182×*APOE* Haplotype	0.152286	0.110879	1.373	0.17
		*4*	89835399	C	G	rs7681440	rs7681440–*APOE* Haplotype	0.070889	0.100602	0.705	0.481

Abbreviations: PD, Parkinson’s disease; DLB, dementia with Lewy bodies; iRBD, idiopathic rapid eye movement (REM) sleep behaviour disorder.

To further explore all potential genetic interactions between *APOE* and *SNCA*, we expanded the range to include all SNPs in *APOE* and *SNCA* plus 200 kb outside the region ends on both genes across all cohorts. We pruned SNPs that were in LD with an *r*^2^ of 0.5 with 316 SNPs remaining in PD, 421 SNPs remaining in DLB and 198 SNPs remaining in iRBD. We applied PLINKs regression-based approach to model and test SNP×SNP interactions. After correction for multiple comparisons with a Bonferroni correction, we did not identify any interactions associated with any of the alpha-synucleinopathies (Supplementary Tables 1–3).

Lastly, a MB-MDR method was implemented as a final screen strategy to detect any significant SNP×SNP and SNP×SNP×SNP interactions using the open-source MB-MDR software.^22^ Here too, no significant interactions were found to be associated with PD, DLB or iRBD after correction for multiple testing using permutation testing across all three cohorts (Supplementary Tables 4–6).

## Discussion

Our results do not support a role for genetic interactions between *APOE* and *SNCA* across PD, DLB and iRBD. The genetic variants that were tested influence the expression and function of these proteins, but it is unlikely that any interactions between them affect the risk of developing synucleinopathies. Although functional epistasis, in the form of biomolecular interaction, can determine biological pathways of disease progression, it may not always be detected through mathematical or statistical genetic interaction analysis. However, if longitudinal disease progression data is part of the analysis, then mixed effects models could be used to analyse within and between subject variability on the interplay between haplotype, *SNCA* risk variants and disease progression. The genetic interactions identified in our study, which represent three synucleinopathies, do not modify the risk of developing PD, DLB or iRBD in our clinical cohorts. This suggests that the pathological interactions between *APOE* and *SNCA* observed in model organisms or human synucleinopathies may not be driven by genetic epistasis influencing disease susceptibility. Therefore, these pathological associations may be driven by other mechanisms such as regulatory factor expression and/or post-translational modifications, protein–protein interactions or environmental factors.

Previous studies have shown that APOE ε4 is linked to DLB in both AD and non-AD cases.^23,24^ However, some studies have also shown that APOE ε4 is only associated with DLB when there is a significant amount of co-existing Alzheimer’s pathology.^25-27^ This finding contradicts the idea that APOE ε4 independently drives α-synuclein pathology.

This study has several limitations. The lack of pathological confirmation of Lewy bodies and AD pathology in the cohorts does not allow for an analysis based on co-pathology. Such analysis would have been able to detect interactions that exist only in subpopulation of patients, for example, those who have both alpha-synuclein and amyloid pathology. Another limitation is that the study scope was limited to *APOE* and *SNCA*. Recently, a stratified GWAS of DLB uncovered an association only between *GBA* rs2230288 and pathologically confirmed DLB without AD pathology, but not in mixed pathological cases.^27^ This same SNP has been identified as a significant risk variant in the most recent GWAS of DLB.^7^ It is possible that large-scale GWAS would uncover associations that encompass a broad spectrum of disease (e.g. DLB with no AD pathology, DLB with mixed PD and AD pathology, etc.), even though these associations may be driven by subsets of these genetic loci (e.g. *GBA*). Furthermore, it is plausible that different disease subtypes consist of distinct genetic combinations and interactions that have not yet been identified at a population level due to the limited size of current sample cohorts. Further studies should include more samples and implement alternative statistical or interrogative methods to leverage the current data to its fullest potential despite its small sample size.

## Supplementary Material

fcaf455_Supplementary_Data

## Data Availability

The data that support the findings of this study are available from dbGaP. Parkinson’s patients and controls (phs000918.v1.p1). DLB patients and controls are available from dbGaP (phs001963.v1.p1). iRBD patients and controls are available upon reasonable request from the corresponding author. Additional controls were also obtained from dbGaP; NGRC (phs000196.v2.p1) and NINDS (phs000089). Code used for analysis can be found on GitHub (https://github.com/gan-orlab/APOE_SNCA).
